# Impact of nirsevimab universal prophylaxis on RSV bronchiolitis hospitalizations. A tertiary level children's hospital perspective

**DOI:** 10.3389/fped.2026.1676689

**Published:** 2026-02-19

**Authors:** Stefania Tonetto, Carolina Cason, Elena Fasiolo, Margherita Dal Cin, Giorgio Cozzi, Manuela Giangreco, Cristina Zappetti, Silvia Nider, Alessandro Amaddeo, Manola Comar, Laura Travan

**Affiliations:** 1 Department of Medical, Surgical and Health Sciences, University of Trieste, Trieste, Italy; 2 Department of Advanced Translational Microbiology, Institute for Maternal and Child Health, IRCCS “Burlo Garofolo”, Trieste, Italy; 3 Department of Health Prevention, Azienda Sanitaria Universitaria Giuliano Isontina, Trieste, Italy; 4 Institute for Maternal and Child Health-IRCCS “Burlo Garofolo”, Trieste, Italy; 5 Central Directorate for Health, Social Policies, Friuli Venezia Giulia Region, Trieste, Italy; 6 Department of Medical Sciences, University of Trieste, Trieste, Italy

**Keywords:** bronchiolitis, hospitalisation, infant, nirsevimab, prophylaxis

## Abstract

**Introduction:**

The 2024–2025 winter season was the first in which Nirsevimab was adopted as universal prophylaxis for bronchiolitis in Friuli Venezia Giulia, a region in the northeastern Italy. This report describes the impact of Nirsevimab universal prophylaxis on bronchiolitis from the perspective of a tertiary-level, children's hospital, the Institute IRCCS Burlo Garofolo in Trieste, Italy.

**Methods:**

We conducted a retrospective observational study reviewing the medical records of all children diagnosed with bronchiolitis during the 2024–2025 winter season, and the winter seasons of the seven preceding years. For each infant admitted, we collected data on age, gender, viral testing result, the need for ventilatory support, and the length of hospital stay. The primary outcome was the number of infant admissions for bronchiolitis during the 2024–2025 winter season, compared with previous years.

**Results:**

During the study period, from 2016 to 2025, 695 infants were diagnosed with bronchiolitis, and 195 were hospitalized. In the 2024–2025 winter season, 597 neonates, 94% of the children born at the Institute, received Nirsevimab prophylaxis. Following the introduction of Nirsevimab, we observed a drastic decrease in the number of infants requiring hospitalization, a marked reduction in infants needing ventilatory support, and a considerable decrease in the cumulative length of hospital stay for bronchiolitis, compared to previous years. These results were clearly related to a substantial decrease in the number of RSV-positive infants arrived at the Institute.

**Discussion:**

In our population, Nirsevimab prophylaxis was very effective and led to a considerable reduction in the number of infants infected with RSV and requiring hospitalization. The hospital burden of bronchiolitis was significantly reduced.

## Introduction

Bronchiolitis is the most common lower respiratory tract infection among neonates and infants in their first year of life, and it is mainly caused by the human respiratory syncytial virus (RSV). RSV is responsible for over 30 million cases of lower respiratory tract infections in preschool children, leading to approximately 3.2 million hospitalizations and 200,000 deaths worldwide each year (1).

Nirsevimab, a new monoclonal antibody specifically targeting the fusion (F) protein of RSV has been developed in the last few years (2, 3). Nirsevimab provides immediate passive immunity through a single intramuscular injection, offering protection for at least 150 days (4).

The European Medicines Agency (EMA) and the Food and Drug Administration (FDA) have approved the use of Nirsevimab in neonates and infants born during or entering their first RSV epidemic season, and in children up to 24 months of age who remain vulnerable to severe RSV disease through their second RSV season.

In Italy, RSV shows annual epidemics in the autumn-winter season, from November to March.

In Friuli Venezia Giulia (FVG), a region in the northeast of Italy, a universal prophylaxis with Nirsevimab for neonates and infants has been approved starting from the 2024–2025 autumn-winter season.

At the tertiary level, university teaching, children's hospital, Institute for Maternal and Child Health IRCCS Burlo Garofolo of Trieste, the capital city of FVG, the distribution of Nirsevimab began on November 4th, 2024 and ended on March 31st, 2025. The monoclonal antibody was offered to every newborn during their neonatal nursery admission. Moreover, Nirsevimab was distributed by the city's territorial healthcare clinics to infants born from April 2024.

The efficacy of nirsevimab in preventing RSV disease has been demonstrated in multiple clinical trials and further supported by a recent systematic review and meta-analysis of real-world evidence (5). Within this framework, the aim of this report was to describe the impact of universal prophylaxis on RSV bronchiolitis from a children's hospital perspective.

## Methods

We conducted a retrospective observational study according to the STROBE guidelines. We reviewed the medical records of all children who received a diagnosis of bronchiolitis, from November 1st, 2016 to March 31st, 2025, at the Institute according to the International Classification of Diseases tenth Revision (ICD-10), codes J21.0, J21.1, J21.8 and J21.9. Data were collected from the ED electronic medical records.

We selected only neonates and infants from zero to 11 months of age, who were diagnosed in the winter season, namely from November to March. Children older than eleven months and/or who received a diagnosis of bronchiolitis in other seasons were excluded.

For each admitted patient, the following data were recorded: age, gender, positivity of the nasal swab for RSV, need for ventilatory support (including High Flow Nasal Cannula, Continuous Positive Airway Pressure, and Non-Invasive Ventilation), and length of hospitalization.

Furthermore, we reviewed the results of PCR-based viral testing performed on nasal swabs in infants, to estimate the prevalence of RSV positivity during the study period.

Data of enrolled infants were summarized using descriptive analysis. Categorical variables were reported as absolute frequencies and percentages, and continuous variables as median and interquartile range (IQR).

We divided the study population into 9 periods according to the patients' arrival dates, from the 2016–2017 to the 2024–2025 autumn-winter season. The interrupted time series (ITS) analysis was also performed on the number of bronchiolitis cases and related hospital admissions by month, with November 2024 designated as the point of interruption. After testing for autocorrelation between the time points of the series, given the presence of autocorrelation at a lag of 12, seasonality was considered. Statistical significance was considered for *p*-values < 0.05. The Kruskal–Wallis test was performed on the number of patients needing ventilatory support and on the length of hospitalization. SAS 9.4 software (SAS Institute Inc., Cary, NC, USA) was used to conduct all statistical analyses.

The primary study outcome was the number of infants who were admitted with a diagnosis of bronchiolitis during the 2024–2025 winter season compared to the 8 previous autumn-winter seasons. Secondarily, we described the number of admitted infants who tested positive for RSV, the number of admitted infants younger than 2 months of age, the number of infants requiring ventilatory support, and the cumulative length of hospital stay related to bronchiolitis in each season.

The Institute's Institutional Review Board (IRB) granted ethical approval to the study protocol (RC 49/22).

## Results

From November 1st, 2024 to March 31st, 2025, 597 neonates were born at the Institute, and 564 (94%) accepted the prophylaxis and received Nirsevimab. No infants received Palivizumab prophylaxis.

During the autumn-winter seasons from 2016 to 2025, 695 infants received a diagnosis of bronchiolitis and 195 (28%) required hospitalization.

**Figure 1:**
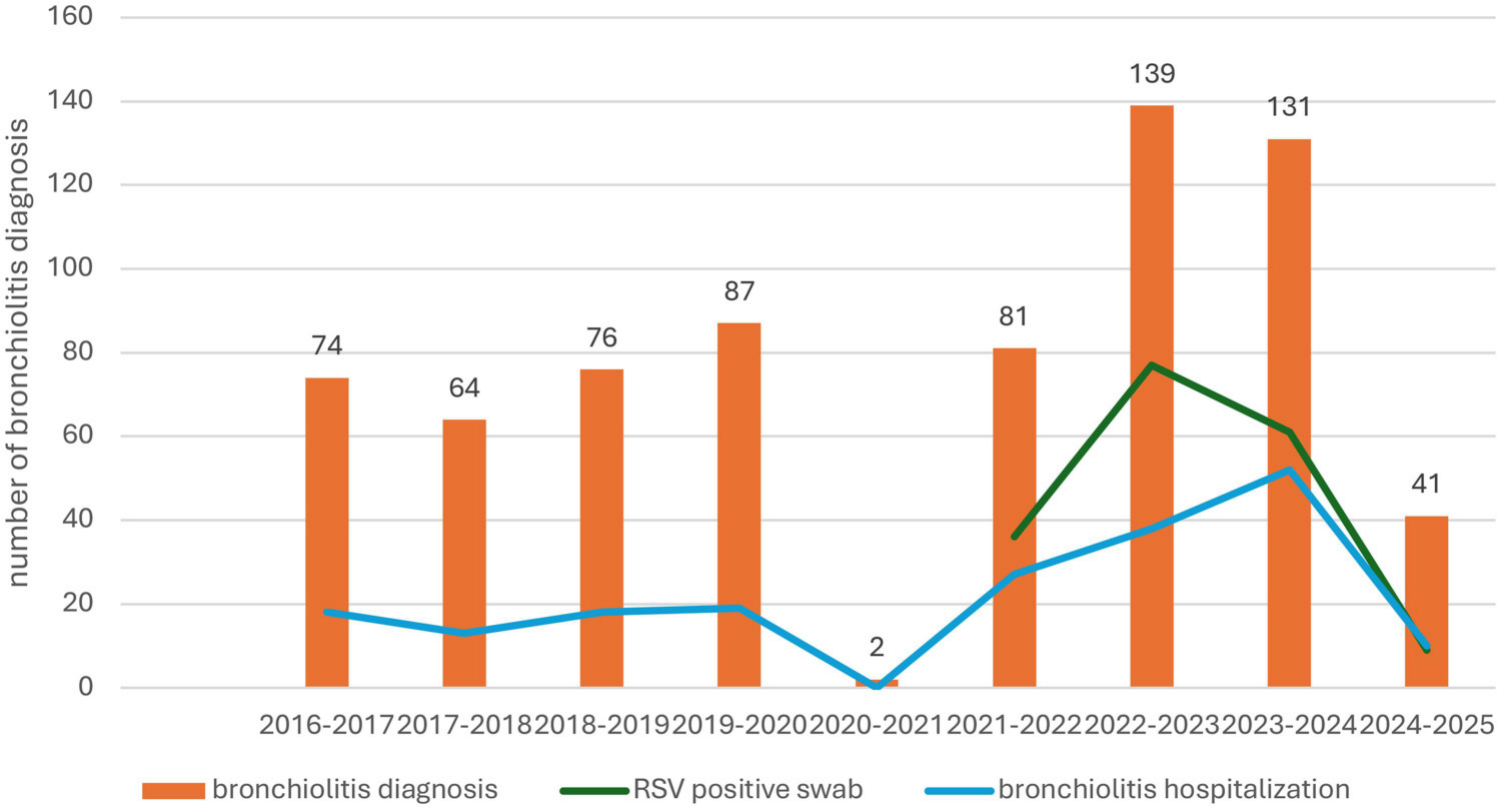
Number of cases of bronchiolitis in the study period. Data regarding RSV molecular testing were available from the 2020-2021 winter season. The exact Cochran-Armitage Trend Test *p*-value: 0.01.

Figure 1 shows the distribution of the cases of bronchiolitis during the study period. The exact Cochran-Armitage Trend Test *p*-value was 0.001. The ITS model estimated a significant (*p* = 0.04) pre-prophylaxis trend with monthly diagnoses increasing by about 0.3%. After controlling for this trend, there was a significant (*p* < 0.0001) level change following the prophylaxis, with monthly diagnoses decreasing by about 20.9% between pre- and post-prophylaxis periods.

Table 1 shows the main study results.

**Table 1:** Main study results.

Characteristics	2016–2017	2017–2018	2018–2019	2019–2020	2020–2021	2021–2022	2022–2023	2023–2024	2024–2025
All infants aged 0–11 months who accessed ED during each respective season (*n*)	2,189	2,099	2,271	2,010	945	1,671	2,161	2,329	1,772
Neonates born between November 1 and March 31, (*n*)	618	687	574	576	588	587	608	574	597
Bronchiolitis diagnosis (*n*, % related to the number of infants who accessed the ED)	74 (3.4)	64 (3.0)	76 (3.3)	87 (4.3)	2 (0.2)	81 (4.8)	139 (6.4)	131 (5.6)	41 (2.3)
Hospital admission for bronchiolitis (*n*, % related to the number of infants who accessed the ED)	18 (0.8)	13 (0.6)	18 (0.8)	19 (0.9)	0	27 (1.6)	38 (1.8)	52 (2.2)	10 (0.6)
Age of admitted patients: (IQR), months	3 (2–5)	2 (1–4)	1 (0–2)	2 (1–3)	0	2 (0–4)	1.5 (0–5)	2 (1–4)	4 (3–7)
Gender male (*n*; % related to the number of bronchiolitis hospital admission)	9 (50)	7 (54)	9 (50)	8 (42)	0	15 (55)	21 (55)	25 (48)	7 (70)
Need for ventilatory support (*n*; % related to the number of hospital admissione for bronchiolitis)	13 (72)	11 (84)	18 (94)	16 (84)	0	25 (93)	35 (92)	42 (81)	6 (60)
Total length of stay for bronchiolitis, days	152	67	129	154	0	155	214	246	47
Hospitalization for RSV bronchiolitis	na	na	na	na	0	16	26	45	5
% RSV positive molecular testing in infants who accessed the ED	na	na	na	na	na	36	77	61	9

Demographic and clinical characteristics of infants hospitalized for bronchiolitis across nine winter seasons. Data are presented as *n* (%) unless otherwise specified. RSV, respiratory syncytial virus; IQR, interquartile range; ED, emergency department; na, not available.

In the 2024–2025 winter season 10 infants were admitted with the diagnosis of bronchiolitis, and this value was significantly lower compared to previous years (*p* = 0.01). A significant (*p* = 0.001) pre-prophylaxis trend emerged with monthly hospital admissions increasing by about 0.3%. After introducing the prophylaxis, monthly hospital admissions decreased significantly (*p* < 0.001) by about 8.7% compared to the pre-prophylaxis period. Among these 10 admitted infants, 5 (50%) tested positive for RSV. Of these 5 RSV-positive infants with bronchiolitis, one had received Nirsevimab. Therefore, in our population, 0.2% (1/564) of neonates who received Nirsevimab prophylaxis developed RSV-positive bronchiolitis requiring hospitalization during their first autumn-winter season.

In the 2024–2025 autumn-winter season 6 infants required ventilatory support, considerably less compared to previous years (*p* < .0001). Similarly, the cumulative hospital length of stay for bronchiolitis, 47 days, was substantially shorter compared to previous years (*p* < .0001).

## Discussion

During the first autumn-winter season with Nirsevimab prophylaxis, we observed a drastic reduction in the number of infants admitted for bronchiolitis, compared to previous years. Moreover, as illustrated in the figure, bronchiolitis diagnoses declined in comparison to both the post-pandemic period—marked by an RSV rebound epidemic (6, 7) and the pre-pandemic years, when annual hospitalization rates for bronchiolitis were largely stable. The decrease was clearly related to the low number of infants infected with RSV. Furthermore, we observed a shift toward an older median age of admitted infants, with only one admitted infant younger than 2 months. This was a notable result considering that neonates and young infants are a population at high risk of severe bronchiolitis. Overall, the hospital burden related to bronchiolitis was substantially reduced in terms of the need for ventilatory support, and the cumulative length of hospital stay for this disease.

Evidence regarding the effectiveness of Nirsevimab prophylaxis in real-world settings is still limited. However, our results are consistent with previous experimental studies (8–10), and align with a Spanish experience (9) that demonstrated a significant reduction of RSV bronchiolitis in terms of hospitalization and intensive care needs. Nevertheless, we cannot exclude the possibility that the widespread use of RSV-specific PCR testing and increased clinical attention since 2021 may have led to an overestimation of bronchiolitis diagnoses among infants admitted with ARIs, directly linked to test positivity.

As previously mentioned, in our city, Nirsevimab prophylaxis was offered both in hospital and in territorial healthcare clinics. This may have increased the acceptance of the offer, thereby strengthening the effectiveness of this measure.

Nirsevimab is not the first pharmacological prophylaxis developed for bronchiolitis. In recent years, Palivizumab, a monoclonal antibody targeting the A antigenic site of the RSV F protein, has been used, but it has been reserved for neonates and infants at high risk of severe disease. Consequently, the population eligible for Palivizumab represented approximately 4%–6% of the pediatric population, limiting its effectiveness in preventing RSV-related hospitalizations (11).

This study has several limitations. Due to the retrospective design, some cases may have been missed or misclassified. Viral spread during the 2020–2021 winter season was completely altered by social distancing measures implemented to combat the SARS-coV-2 pandemic (12, 13), so we excluded that season from statistical comparisons. Prior to the SARS-coV-2 pandemic, our Institute's policy did not routinely mandate viral testing for infants admitted with bronchiolitis, preventing us from quantifying the impact of RSV on hospitalizations during that period. Moreover, we restricted our study period to autumn-winter seasons, potentially overlooking out-of-season RSV outbreaks. Additionally, we did not account for the potential impact of SARS-coV-2 infection control measures on the circulation of RSV. However, we considered this potential bias to be negligible, given the absence of significant infection control practices or other public health measures during the most recent winter season. Moreover, we did not compare the proportion of hospitalized infants who had received Palivizumab prophylaxis in previous years with those who received Nirsevimab this season. However, the number of infants who received Palivizumab at our Institute was limited to very specific cases (11).

We collected data regarding general hospital admissions, but not to the intensive care unit. Therefore, we were unable to estimate the effect of Nirsevimab prophylaxis on intensive care admissions for bronchiolitis.

Furthermore, our data were collected from a single Institute, limiting generalizability. In this sense, a Spanish study showed a different distribution of bronchiolitis cases before and after the COVID-19 pandemic, compared to our observations (14). Finally, cost-effectiveness considerations and prevalence of RSV infection impact on outpatient services or community were not addressed, as they were beyond the scope of this study. However, it should be noted that targeted cost-effectiveness studies have yielded conflicting results (15–17), and the perceived benefits of immunization might be overestimated due to the atypical RSV rebound epidemics observed in the post-pandemic period. Additional research will be required to better understand these issues.

In conclusion, in our population, Nirsevimab prophylaxis was very effective, and led to a drastic reduction in the number of infants infected with RSV and requiring hospitalization. After introduction of Nirsevimab, the hospital burden caused by bronchiolitis significantly decreased, falling below both post-pandemics rebound levels and the previously stable pre-pandemic annual trends.

These encouraging findings support the widespread implementation of this preventive measure to further reduce the burden of bronchiolitis on healthcare systems.

## Data Availability

The raw data supporting the conclusions of this article will be made available by the authors, without undue reservation.
